# Establishment of KGAS, a cell line derived from gastric-type adenocarcinoma of the uterine cervix

**DOI:** 10.1007/s13577-025-01286-9

**Published:** 2025-09-15

**Authors:** Hiroaki Yamada, Akira Yokoi, Eri Asano-Inami, Masami Kitagawa, Kosuke Yoshida, Kazuhiro Suzuki, Shin Nishio, Hiroaki Kajiyama, Naotake Tsuda

**Affiliations:** 1 Department of Obstetrics and Gynecology, Nagoya University Graduate School of Medicine, 65 Tsurumai-Cho, Showa-Ku, Nagoya, 466-8550 Japan; 2 Nagoya University Institute for Advanced Research, Furo-Cho, Nagoya, 464-8601 Japan; 3 Department of Obstetrics and Gynecology, Kurume University School of Medicine, 67 Asahimathi, Kurume, 830-0011 Japan

**Keywords:** Cervical cancer, Gastric-type adenocarcinoma of the uterine cervix, Chemoresistance, Patient-derived cell line

## Abstract

Gastric-type adenocarcinoma (GAS) of the uterine cervix is a rare and aggressive subtype of cervical adenocarcinoma characterized by intrinsic resistance to chemotherapy and poor clinical outcomes due to the lack of effective treatment options. To address this critical unmet need, we established a novel GAS-derived cell line, KGAS, from ascitic fluid collected from a patient with recurrent GAS. Short tandem repeat (STR) analysis confirmed the genetic identity between the primary tumor and the cell line. Upon transplantation into immunocompromised mice, KGAS cells formed tumors that expressed Claudin-18 and MUC6, clinically recognized markers of GAS. Furthermore, KGAS cells exhibited marked resistance to paclitaxel and carboplatin, showing significantly reduced growth inhibition compared to HeLa cells. We also established a paclitaxel- and carboplatin-resistant subline, rKGAS, and performed microRNA (miRNA) sequencing to explore the molecular basis of acquired chemoresistance. Seventeen differentially expressed miRNAs were identified between KGAS and rKGAS cells. Upregulated miRNAs in rKGAS were predicted to target oncogenes such as BCL2, MET, SIRT1, and VEGFA, whereas downregulated miRNAs were associated with tumor suppressor genes, including IGF1R, TNFAIP3, and MTOR. The KGAS and rKGAS cell lines represent valuable preclinical models for elucidating the molecular mechanisms of chemoresistance and malignant progression in cervical GAS, and may contribute to the development of novel therapeutic strategies for this challenging cancer subtype.

**Supplementary Information:**

The online version contains supplementary material available at 10.1007/s13577-025-01286-9.

## Introduction

Cervical cancer is the fourth most common cancer in women worldwide, with approximately 570,000 new cases diagnosed and 310,000 deaths reported annually [1]. Due to the widespread adoption of human papillomavirus (HPV) vaccination, the incidence of HPV-dependent cervical cancers is decreasing. However, the relative proportion of HPV-independent cervical cancers is expected to increase [2]. While squamous cell carcinoma accounts for the majority of cervical cancers, adenocarcinoma comprises about 20%, among which gastric-type adenocarcinoma of the uterine cervix (GAS) represents a rare subtype, accounting for approximately 10% of adenocarcinomas [3]. GAS was first defined in 2007 by Kojima et al. as “an adenocarcinoma characterized by well-defined cell borders and abundant clear or pale eosinophilic cytoplasm.” [4] This type of cancer is known for its diagnostic challenges and resistance to treatment, with poorer progression-free survival (PFS) and overall survival (OS) compared to usual-type endocervical adenocarcinoma [5]. Moreover, 80% of GAS cases are well-differentiated, making diagnosis through conventional cytology and biopsy difficult [4]. GAS was newly classified as an HPV-independent subtype in the WHO 2020 classification, distinguishing it from HPV-associated cervical adenocarcinomas [6].

Despite its distinct histological and molecular features, no patient-derived cell lines of GAS have been reported to date for use as research models. This lack of models poses a significant obstacle to elucidating the pathophysiology of GAS and developing novel therapeutic strategies. In this study, we successfully established a patient-derived GAS cell line and analyzed its biological characteristics and drug sensitivity. This novel cell line offers a promising platform for elucidating the molecular mechanisms of GAS and advancing new therapeutic strategies.

## Materials and methods

### Patients

**Fig. 1:**
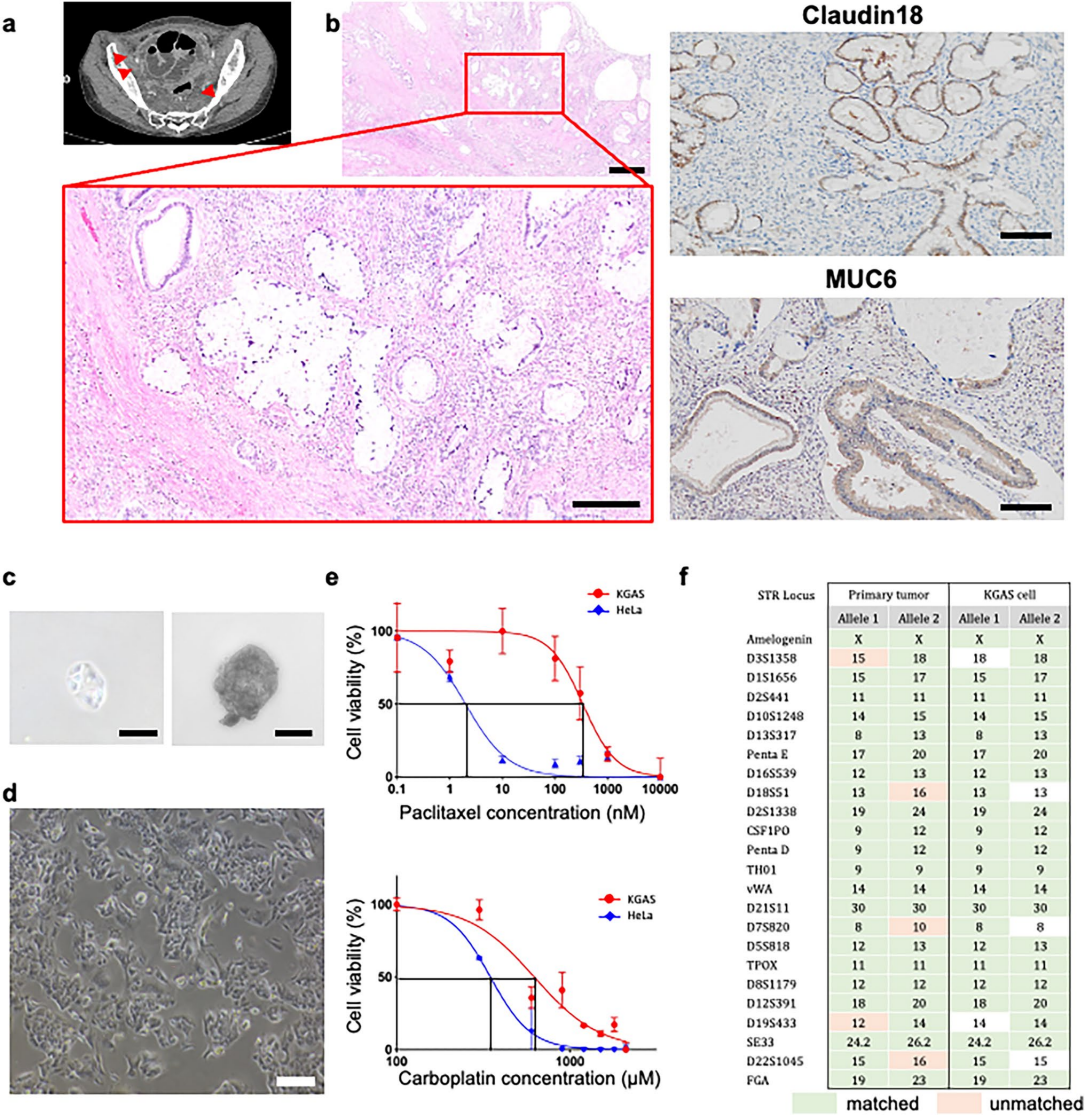
Clinical and pathological features and establishment of the KGAS cell line. **a** Abdominal Computed Tomography Image obtained after ascitic fluid puncture, showing significant fluid accumulation in the pelvic cavity. Arrows indicate peritoneal dissemination and ascites accumulation. **b** Histopathological image of the primary tumor from the initial surgery (Hematoxylin and Eosin staining). Scale bars: (top panel) 500 µm, (bottom panel) 100 µm. Immunohistochemical staining of the primary tumor shows positive expression of Claudin-18 and MUC6, consistent with gastric-type differentiation. Scale bar: 100 µm. **c** Representative images of single-cell-derived spheres formed by KGAS cells in low-attachment culture, indicating self-renewal ability. Scale bars: (left) 10 µm; (right) 50 µm. **d** Phase-contrast image of the established KGAS cell line. Scale bar: 100 µm. **e** Cell viability of KGAS and HeLa cells following 48-h exposure to paclitaxel (top) and carboplatin (bottom). KGAS cells showed significantly lower sensitivity compared to HeLa cells. Data are presented as mean ± Standard Deviation of n = 3 independent experiments, each performed with technical replicates. Passage numbers: KGAS (passages 9–15), HeLa (passages 10–15). **f** Short Tandem Repeat (STR) analysis of the primary tumor and KGAS cell line. STR profiles matched at 89.13% across 21 loci and amelogenin, confirming derivation from the same patient

The patient was 42 years old and had cervical cancer (Stage IIB, FIGO2018) with a gastric-type adenocarcinoma. As a surgical treatment, a radical hysterectomy was performed. Five months after the initial treatment, recurrence was observed in the vaginal stump and pelvic lymph nodes, necessitating radiotherapy. However, there was no therapeutic effect, and she developed carcinomatous peritonitis with peritoneal dissemination and ascites accumulation (Fig. 1a). Pathological examination revealed adenocarcinoma primarily involving the uterine cervix, showing destructive, infiltrative growth throughout the full thickness of the cervical tissue. The tumor exhibited diverse histological patterns, including glands with minimal structural atypia, complex branching glandular proliferation, sieve-like glandular structures, and poorly differentiated infiltrative growth. The cell borders were well-defined, and the nuclei displayed a spectrum of atypia, ranging from inconspicuous forms to those with prominent nucleoli and coarse chromatin patterns (Fig. 1b). Immunohistochemical staining revealed MUC2 negative, Claudin18 positive, MUC6 positive, p53 positive, and p16 negative, collectively confirming gastric-type differentiation and supporting the diagnosis of GAS. After surgery, a total of six cycles of tri-weekly paclitaxel (PTX) and carboplatin (CBDCA) were administered as adjuvant chemotherapy. The protocol for establishing the following cell line was approved by the Ethics Committee of Kurume University Hospital, and written informed consent was obtained from the patient (approval number 22131).

### Patient-derived tumoroid culture

Under sterile conditions, 1 L of ascitic fluid was collected from a patient with carcinomatous peritonitis. This fluid was centrifuged at 1500 rpm for 10 min to isolate the suspended cancer cells. The resulting cell pellet was washed three times with phosphate-buffered saline to remove contaminants. Cells were then cultured in a low-serum medium containing 5% fetal calf serum supplemented with antibiotics (100 U/mL penicillin and 100 µg/mL streptomycin) at 37 °C in a humidified atmosphere containing 5% CO₂. During the first week of culture, the medium was changed every 2–3 days under aseptic conditions to maintain cell viability and remove debris. Cultured cells were monitored daily under a phase-contrast microscope to observe growth and morphological changes, and a GAS cell line was established from the cultured cells. To generate a chemotherapy-resistant cell line, KGAS cells were exposed to PTX (15 nM) and CBDCA (15 µM). Drug solutions were freshly prepared and added to the culture medium weekly for 24 h. After each 24-h drug treatment, culture medium was replaced with a drug-free medium to allow recovery. This process was repeated weekly for 6 weeks. During this period, the cells were monitored for morphological changes under a phase-contrast microscope. Only viable cells that survived post drug exposure were continuously cultured under the same conditions. When the resistant cells exhibited stable proliferation across multiple passages, the cell line was designated as rKGAS.

### Cell line authentication

To confirm the uniqueness and authenticity of the established cell line KGAS, we performed STR profiling using the PowerPlex® Fusion 6 C system (Promega). Genomic DNA was extracted from the original tumor, KGAS, and rKGAS cells, and STR analysis was conducted on 21 autosomal loci and the amelogenin sex-determining marker. The STR profiles of KGAS and rKGAS matched the original tumor DNA with 89.13% similarity, which is consistent with derivation from the same donor based on ICLAC criteria. Furthermore, comparison with publicly available STR profiles using the ATCC STR database (https://www.atcc.org/str-database) showed a maximum match percentage of only 63% for any known cell line. This indicates no cross-contamination and strongly supports the originality of KGAS. Importantly, STR analysis of rKGAS cells also demonstrated the same 89.13% match with the original tumor DNA, confirming their derivation from the same patient source (Fig. S1a).

### Sphere formation assay

To evaluate the sphere-forming ability, a functional hallmark of cancer stem-like cells, single KGAS cells were seeded into 96-well ultra-low attachment plates using the limiting dilution method. Cells were cultured in DMEM supplemented with 5% FCS, without the addition of exogenous growth factors. Given that KGAS cells exhibited a protein expression profile indicative of a cancer stem cell phenotype, we assessed their self-renewal capacity by monitoring sphere formation in wells confirmed to contain single cells. Spheres were observed in all wells (n = 23) that had been confirmed to contain single cells, suggesting a strong self-renewal capacity under these conditions.

### Establishment of CDX mouse model

Eight-week-old female BALB/cSlc-nu/nu mice were purchased from Japan SLC, Inc. (Shizuoka, Japan). To evaluate the tumorigenic potential of KGAS cells, luciferase-expressing KGAS cells (Luc-KGAS; 1.0 × 10⁷ cells suspended in 100 μL of a 1:1 mixture of PBS and Matrigel) were injected subcutaneously into both flanks of the mice. Tumor formation was monitored twice weekly using in vivo imaging, and visible tumors typically developed within 3–4 weeks. The excised tumors were fixed in formalin, embedded in paraffin (FFPE), and subjected to hematoxylin and eosin (H&E) staining. All animal procedures were reviewed and approved by the Nagoya University Institutional Animal Experimentation Committee (Approval No. 240332). In addition, they complied with the ARRIVE guidelines and were carried out in accordance with the U.K. Animals (Scientific Procedures)Act, 1986, and related guidelines.

### MTS assay

Cell proliferation was assessed using the CellTiter 96® AQueous One Solution Cell Proliferation Assay (Promega, Madison, WI), following the manufacturer’s instructions. KGAS, rKGAS, and HeLa cells were seeded in 96-well plates at a density of 5 × 10^3^ cells per well and incubated overnight. HeLa cells were purchased from RIKEN BRC (Tsukuba, Japan) and cultured in Dulbecco’s Modified Eagle Medium (DMEM) supplemented with 10% fetal bovine serum (FBS) at 37 °C. To evaluate their antiproliferative effects, cells were treated with PTX or CBDCA at various concentrations. For PTX, KGAS and HeLa cells were treated with a broad range of concentrations (0.01, 0.1, 1, 10, 100, 300, 1000, and 10,000 nM), while KGAS and rKGAS cells were treated with a narrower range (100, 200, 300, 400, 500, 600, 700, and 1000 nM), based on preliminary experiments and observed sensitivity. For CBDCA, the concentrations used were consistent for all cell types (KGAS, rKGAS, and HeLa), ranging from 100 to 2100 µM (100, 300, 600, 900, 1200, 1500, 1800, and 2100 µM). Cell viability was assessed by measuring absorbance at 490 nm, which reflects the number of metabolically active cells. Unless otherwise indicated, experiments were performed using KGAS passages 9–15, rKGAS passages 9–15, and HeLa passages 10–15. Data are presented as mean ± Standard Deviation, and ‘n’ refers to the number of independent experiments (n = 3), each conducted with technical replicates.

### Flow cytometry analysis

The protein expression of the established KGAS was analyzed using a flow cytometer. To confirm the characteristics of GAS, which is resistant to chemotherapy and radiotherapy, we analyzed cancer stem cell markers (ALDH: ALDEFLUOR™ Assay Kit, STEMCELL Technologies; CD44v9: Invitrogen) and epithelial-mesenchymal transition (EMT) markers (EpCAM, Snail1/2/3, ZEB: Bios Antibodies). The experiment was performed once using KGAS and rKGAS cells.

### RNA extraction and miRNA sequencing

Total RNA was extracted from KGAS and rKGAS cell lines, including stimulated cell lines. Small RNA was confirmed to be present at a concentration of 0.5% or more using the BioAnalyzer 2100TM and Small RNA Kit (Agilent Technologies, Palo Alto, Calif.). Next, cDNA libraries were prepared using 50–100 ng of small RNA samples with the Ion Total RNA-Seq Kit v2® (Thermo Fisher Scientific, Waltham, Mass.). The cDNA concentration was measured using the Agilent High Sensitivity DNA Kit® (Agilent Technologies, Palo Alto, Calif.). Next-generation sequencing was performed using the Ion S5TM System (Thermo Fisher Scientific, Waltham, Mass.) with the Ion540TMChip (Thermo Fisher Scientific, Waltham, Mass.). The expression levels of the detected miRNAs were calculated and normalized using RPM (Read Per Million Mapped Reads), and low-expressed miRNAs (< 20 reads in all samples) were excluded from further analyses.

### *In silico* analysis

To analyze the gene interactions of the identified miRNAs, we used miRTargetLink 2.0 (Tokar et al., 2018; https://ccb-compute2.cs.uni-saarland.de/mirtargetlink2/) to visualize experimentally validated miRNA-gene associations.

## Results

### Establishment and chemoresistance of the KGAS cell line

Initially, most cells formed non-adherent spheres, a characteristic associated with tumorigenic potential. Under low-nutrient culture conditions, KGAS cells formed well-defined spheroid structures within 3 to 4 weeks, indicating their self-renewal capacity (Fig. 1c). Over the course of the first week, some spheres began to disaggregate, and individual cells adhered to the culture surface. The adherent cells proliferated and formed a monolayer with uniform morphology. After approximately 10 passages over three months, a stable cell line (KGAS) was established, consisting of large, morphologically homogeneous tumor cells (Fig. 1d). MTS assays were performed to evaluate cell viability following treatment with PTX and CBDCA, which are commonly used chemotherapeutic agents for cervical cancer. Each drug was applied individually, and cell viability was assessed 48 h post-treatment. The IC_50_ value of PTX for KGAS cells was 338.2 nM, markedly higher than that for HeLa cells (2.0 nM). Similarly, the IC_50_ of CBDCA was 621.8 μM in KGAS cells, compared to 347.8 μM in HeLa cells. These findings indicate that GAS (KGAS cells) exhibits greater chemoresistance to both PTX and CBDCA compared to HeLa cells (Fig. 1e).

### Establishment of CDX mouse model

**Fig. 2:**
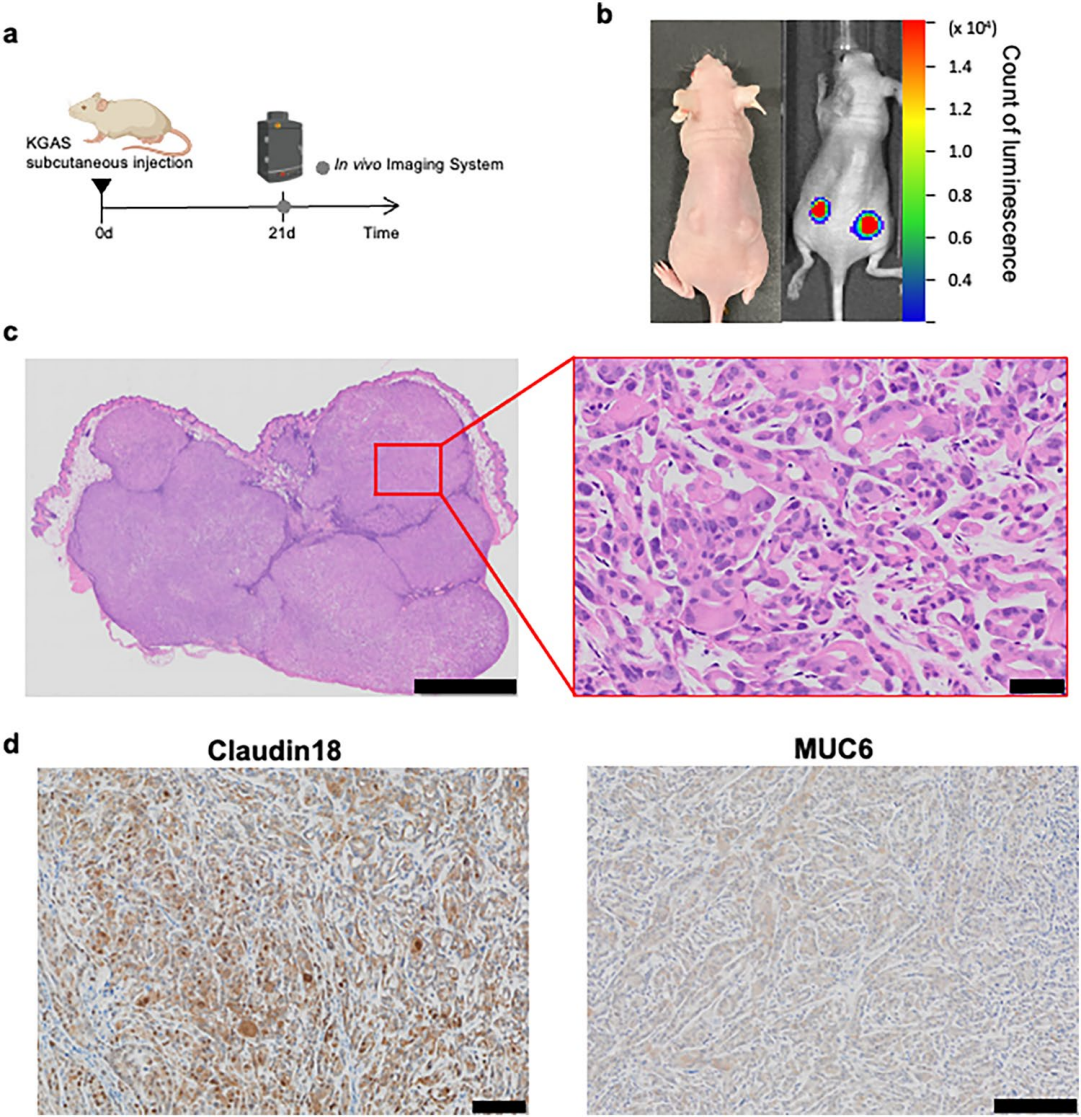
Establishment of CDX mouse model using KGAS cells. **a** Schematic representation of the experimental design. Luciferase-expressing KGAS cells were subcutaneously injected into both flanks of immunodeficient mice, and tumor growth was monitored using an in vivo bioluminescence imaging system. **b** Bioluminescence imaging of tumor-bearing mice 21 days after injection. Luminescent signals at the injection sites confirm tumor formation by KGAS cells. **c** Hematoxylin and Eosin staining of the xenograft tumor. The left panel shows a low-magnification image of a well-formed subcutaneous tumor mass. Scale bar: 2 mm. The right panel shows a high-magnification image highlighting invasive, atypical glandular structures. Scale bar: 50 µm. **d** Immunohistochemical analysis of the xenograft tumor demonstrating positive expression of Claudin-18 (left, Scale bar: 100 µm) and MUC6 (right, Scale bar: 200 µm), indicating the retention of gastric-type differentiation in vivo

To evaluate the tumorigenic potential of KGAS cells in vivo, a CDX mouse model was established using immunodeficient mice (Fig. 2a). Luciferase-expressing KGAS cells were subcutaneously injected into both flanks of the mice. Tumor formation was observed in 18 out of 22 mice. In vivo imaging using the IVIS system confirmed luminescence signals at the injection sites (Fig. 2b). The excised tumors exhibited glandular carcinoma-like structures and were histologically confirmed as tumor tissue by H&E staining (Fig. 2c). Furthermore, immunohistochemical staining of the resected tumors for Claudin18 and MUC6 showed positive results, suggesting that KGAS cells retained a gastric-type adenocarcinoma phenotype even in vivo (Fig. 2d).

### Evaluation of phenotypic changes in rKGAS cells induced by low-dose drug exposure

**Fig. 3:**
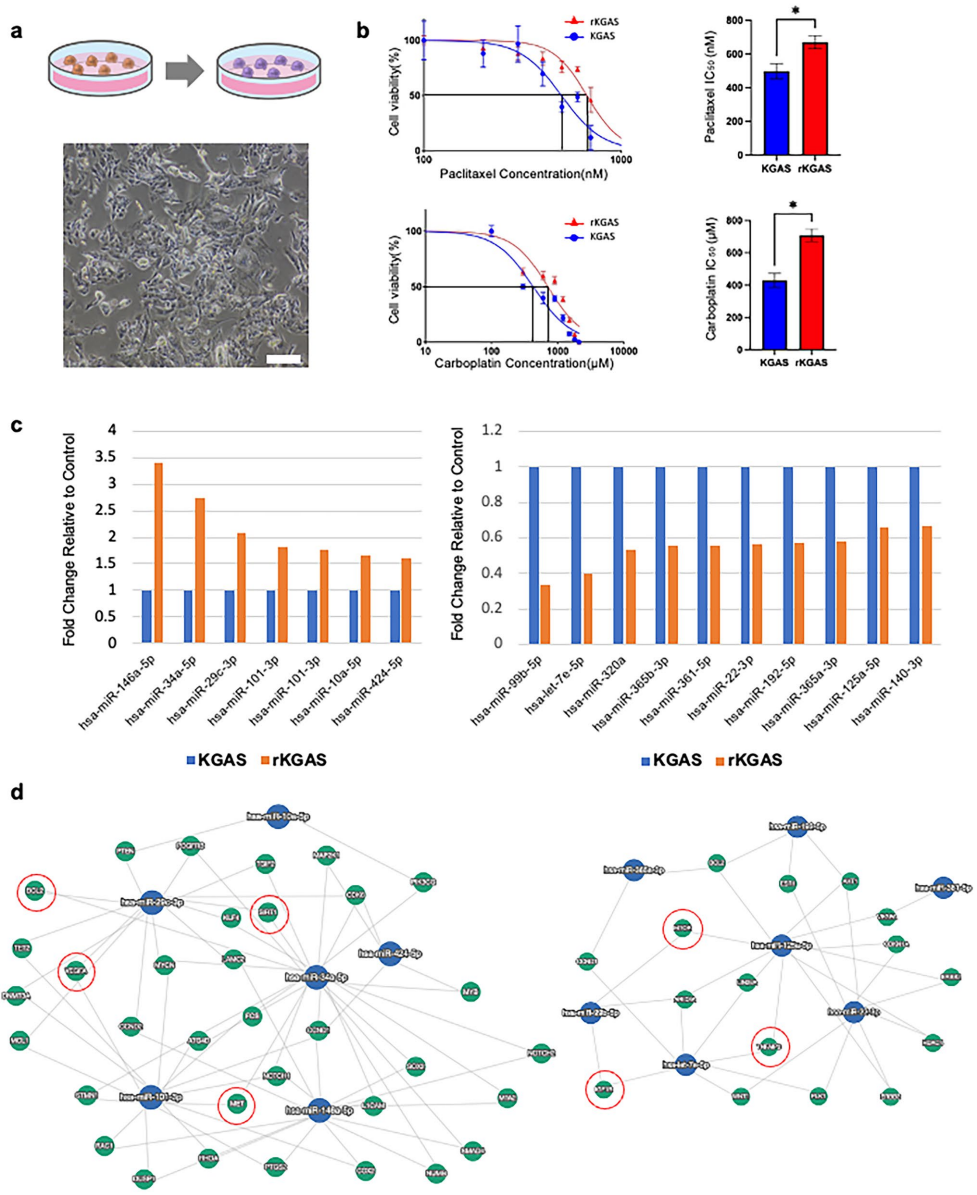
Phenotypic and molecular characterization of chemotherapy-resistant rKGAS cells. **a** Schematic illustration of rKGAS cell generation by repeated exposure to low-dose paclitaxel and carboplatin. The phase-contrast image shows morphological changes in rKGAS cells compared to parental KGAS cells, including increased size and irregular nuclear contours (Scale bar: 100 µm). **b** Cell viability of KGAS and rKGAS cells following 48-h exposure to paclitaxel (top left) and carboplatin (bottom left), assessed by MTS assay. Bar graphs (right) summarize the IC₅₀ values, indicating increased drug resistance in rKGAS cells. Data are presented as mean ± Standard Deviation of n = 3 independent experiments, each performed with technical replicates. Passage numbers: KGAS (passages 9–15), rKGAS (passages 9–15). **c** Upregulated miRNAs in rKGAS (left) and downregulated miRNAs in rKGAS (right) compared to KGAS. **d** Gene-miRNA interaction network visualized by miRTargetLink. Left: miRNAs upregulated in KGAS target multiple oncogenes including *BCL2*, *MET*, *SIRT1*, and *VEGFA* (circled in red). Right: miRNAs upregulated in rKGAS target tumor suppressor genes such as *IGF1R*, *TNFAIP3*, and *MTOR* (circled in red)

The chemoresistant cell line (rKGAS) was established by continuously exposing KGAS cells to low doses of PTX (15 nM) and CBDCA (15 µM). Compared to KGAS cells, rKGAS cells exhibited notable morphological changes, including increased cell size, nuclear irregularities, increased cell layering, and a greater number of floating cells (Fig. 3a). Drug sensitivity was evaluated using the MTS assay. The IC_50_ for PTX was 497.3 nM in KGAS and 669.3 nM in rKGAS, while for CBDCA, it was 424.9 µM in KGAS and 708.2 µM in rKGAS, indicating a modest increase in drug tolerance in rKGAS cells following low-dose drug exposure (Fig. 3b). Furthermore, the expression of markers associated with cancer stemness and epithelial–mesenchymal transition (EMT) was analyzed by flow cytometry in both KGAS and rKGAS cells. EpCAM and ALDH expression levels were decreased in rKGAS cells, whereas CD44v9 expression was increased (Supp. Figure 1b). These findings suggest that the acquisition of chemoresistance may be accompanied by changes in stem cell-like properties and EMT-related characteristics.

### miRNA profiling of differentially expressed miRNAs in rKGAS cells

To identify characteristic pathways associated with differences in miRNA expression between the two cell lines, we analyzed the miRNA profiles of KGAS and rKGAS. RNA was extracted from both cell lines, and miRNA sequencing was performed. After excluding miRNAs with a total read count of less than 20, 89 miRNAs were included in the analysis. Differentially expressed miRNAs with an absolute fold change value greater than 1.5 were identified, including 7 miRNAs with increased expression and 10 miRNAs with decreased expression in rKGAS compared to KGAS, totaling 17 miRNAs (Fig. 3c). Gene interaction analysis demonstrated that miRNAs highly expressed in rKGAS cells commonly targeted key oncogenes, including *BCL2*, *MET*, *SIRT1*, and *VEGFA* (Fig. 3d, left panel). In contrast, miRNAs downregulated in rKGAS cells were found to regulate tumor suppressor genes such as *IGF1R*, *TNFAIP3*, and *MTOR*, suggesting that the acquisition of chemoresistance may involve altered regulation of both oncogenic and tumor-suppressive pathways (Fig. 3d, right panel).

## Discussion

GAS is a rare and aggressive subtype of cervical adenocarcinoma, accounting for approximately 10% of all cases [4, 6]. Despite its clinical significance, basic research on GAS remains limited, primarily focusing on genomic analyses [7]. The lack of progress in understanding GAS can largely be attributed to the absence of established cell lines derived from GAS and the scarcity of in vivo studies exploring its biological characteristics [8]. In this study, we successfully established the KGAS cell line from ascites collected from a patient with recurrent GAS, who initially underwent surgery and TC chemotherapy but later developed peritoneal dissemination and ascites accumulation.

In this study, we established the KGAS cell line as a novel model to investigate the biological characteristics of cervical cancer, with a particular focus on chemoresistance and cancer stem cell-like properties [9]. Our findings demonstrated that KGAS cells exhibit significant resistance to PTX and CBDCA, two chemotherapeutic agents commonly used in cervical cancer treatment [10]. Compared to HeLa cells, the growth inhibition rates of KGAS cells were notably lower, suggesting that KGAS cells harbor unique molecular characteristics contributing to chemoresistance. This resistance reflects a key challenge in cervical cancer therapy and underscores the importance of identifying new therapeutic strategies. The chemoresistant phenotype observed in KGAS cells may be associated with the expression of cancer stem cell (CSC) and epithelial–mesenchymal transition (EMT) markers [11–13]. Flow cytometry analysis revealed that KGAS and rKGAS cells exhibited distinct marker expression profiles, including differential levels of EpCAM, ALDH, and CD44v9 (Fig. S1b). These findings suggest phenotypic changes associated with chemoresistance acquisition [11]. The in vivo tumorigenicity of KGAS cells, demonstrated by the formation of subcutaneous tumors in the CDX mouse model, supports their cancer stem cell-like properties. This ability to initiate tumors in immunodeficient mice fulfills a key functional hallmark of cancer stem cells, suggesting that KGAS cells may harbor a subpopulation with self-renewal and differentiation potential, consistent with our in vitro findings [14].

miRNA profiling of KGAS and rKGAS cells provided further insights into the molecular mechanisms underlying chemoresistance. A total of 17 differentially expressed miRNAs were identified.

miRNAs upregulated in rKGAS cells were found to target oncogenes such as *BCL2*, *MET*, *SIRT1*, and *VEGFA*, which are involved in promoting cell survival, metastasis, and resistance to apoptosis [15–18]. In contrast, miRNAs that were downregulated in rKGAS cells were shown to regulate tumor suppressor genes including *IGF1R*, *TNFAIP3*, and *MTOR*, suggesting a potential loss of tumor-suppressive signaling in rKGAS cells [19–21].

These findings highlight the critical role of miRNA regulation in the acquisition of chemoresistance in cervical cancer cells. The KGAS and rKGAS cell lines serve as valuable platforms for cervical cancer research; however, the limitations of this study must also be acknowledged. First, flow cytometry analysis of CSC and EMT markers was conducted as a preliminary study with limited replicates. Although differences in EpCAM, ALDH, and CD44v9 expression were observed between KGAS and rKGAS cells, further experiments with biological replicates are needed to validate these findings. Second, although miRNA profiling revealed 17 differentially expressed miRNAs, functional validation such as miRNA overexpression or knockdown assays was not performed in this study. Confirming the direct regulatory effects of these miRNAs on their predicted target genes will be essential in future investigations. Additional transcriptomic and genetic analyses will also contribute to a more comprehensive understanding of chemoresistance mechanisms in GAS.

In vitro cell culture models inherently lack the complexity of the in vivo tumor microenvironment, including interactions with stromal cells, immune cells, and vasculature, which significantly influence tumor behavior [20, 22]. To address this limitation, we also established a CDX mouse model using luciferase-expressing KGAS cells. This model not only demonstrated the in vivo tumorigenic potential of KGAS cells but also enabled bioluminescence-based tumor monitoring [23]. The in vivo system provides a critical platform for validating the biological relevance of the KGAS and rKGAS cell lines and serves as a foundation for future studies investigating interactions with the tumor microenvironment [24].

Furthermore, comprehensive genetic analyses, including whole-genome sequencing and transcriptomic profiling, are needed to elucidate the broader molecular landscape of chemoresistance in KGAS cells. We plan to perform exome sequencing and karyotype analysis in future studies to further define the genomic and chromosomal alterations characteristic of GAS.

In conclusion, the establishment of the KGAS and rKGAS cell lines represents a significant advancement in cervical cancer research. These cell lines provide a robust model for investigating the molecular basis of chemoresistance and the role of cancer stem cell-like properties in therapy resistance. Our findings contribute to the growing body of evidence supporting the importance of miRNA regulation and cancer stem cell markers in cervical cancer progression. The KGAS and rKGAS cell lines provide novel tools for understanding the molecular mechanisms of chemoresistance in cervical cancer, thereby facilitating further functional investigation and potentially leading to the development of novel therapies.

## Supplementary Information

Below is the link to the electronic supplementary material.

Supplementary file1 (PDF 84 KB)

## Data Availability

Data are available upon reasonable conditions.
