# Supplementary material for: Establishment of KGAS, a cell line derived from gastric-type adenocarcinoma of the uterine cervix
Source: Hum Cell. 2025 Sep 15;38(6):159. doi: 10.1007/s13577-025-01286-9 (PMC12436518; doi:10.1007/s13577-025-01286-9)
Supplement: Supplementary file 1 — Supplementary file1 (PDF 84 KB) [file 13577_2025_1286_MOESM1_ESM.pdf]

Fig. S1

a

89.13 % 89.13 %

STR Locus	Primary tumor		KGAS cell		rKGAS cell	
	Allele 1	Allele 2	Allele 1	Allele 2	Allele 1	Allele 2
Amelogenin	X	X	X	X	X	X
D3S1358	15	18	18	18	18	18
D1S1656	15	17	15	17	15	17
D2S441	11	11	11	11	11	11
D10S1248	14	15	14	15	14	15
D13S317	8	13	8	13	8	13
Penta E	17	20	17	20	17	20
D16S539	12	13	12	13	12	13
D18S51	13	16	13	13	13	13
D2S1338	19	24	19	24	19	24
CSF1PO	9	12	9	12	9	12
Penta D	9	12	9	12	9	12
TH01	9	9	9	9	9	9
vWA	14	14	14	14	14	14
D21S11	30	30	30	30	30	30
D7S820	8	10	8	8	8	8
D5S818	12	13	12	13	12	13
TPOX	11	11	11	11	11	11
D8S1179	12	12	12	12	12	12
D12S391	18	20	18	20	18	20
D19S433	12	14	14	14	14	14
SE33	24.2	26.2	24.2	26.2	24.2	26.2
D22S1045	15	16	15	15	15	15
FGA	19	23	19	23	19	23

matched

unmatched

b

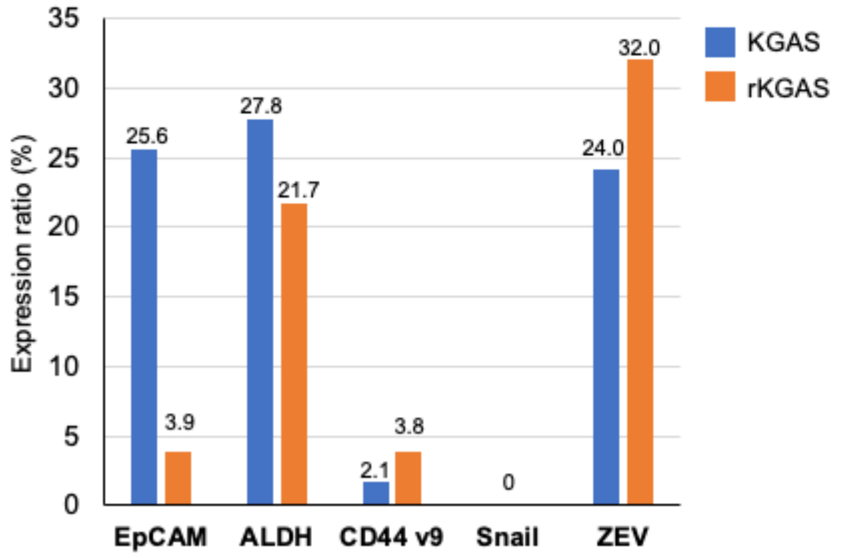

Fig. S1. STR profiling and flow cytometry analysis.
(a) STR electropherogram confirming the genetic identity between the primary tumor, KGAS, and rKGAS cells.
(b) Flow cytometry analysis of KGAS and rKGAS cells.
